# Adding value to real-world data: the role of biomarkers

**DOI:** 10.1093/rheumatology/kez113

**Published:** 2019-04-16

**Authors:** Darren Plant, Anne Barton

**Affiliations:** 1 Manchester Academic Health Science Centre, The University of Manchester, Arthritis Research UK Centre for Genetics and Genomics, Centre for Musculoskeletal Research, UK; 2 Manchester Academic Health Science Centre, NIHR Manchester Biomedical Research Centre, Manchester University NHS Foundation Trust, Manchester, UK

**Keywords:** rheumatoid arthritis, real-world data, registry, biomarkers, precision medicine

## Abstract

Adding biomarker information to real world datasets (e.g. biomarker data collected into disease/drug registries) can enhance mechanistic understanding of intra-patient differences in disease trajectories and differences in important clinical outcomes. Biomarkers can detect pathologies present early in disease potentially paving the way for preventative intervention strategies, which may help patients to avoid disability, poor treatment outcome, disease sequelae and premature mortality. However, adding biomarker data to real world datasets comes with a number of important challenges including sample collection and storage, study design and data analysis and interpretation. In this narrative review we will consider the benefits and challenges of adding biomarker data to real world datasets and discuss how biomarker data have added to our understanding of complex diseases, focusing on rheumatoid arthritis.

**Rheumatology key messages**

- Addition of biomarker information to real-world datasets is challenging but could capture clinically useful information.
- Biomarkers can detect pathologies present early in disease potentially paving the way for preventative interventions.
- Biomarkers can quantify important exposures underpinning intra-patient differences in disease outcomes to facilitate precision medicine.

## Introduction

Real world datasets include information collected from individuals in routine clinical care with a particular condition or diagnosis or receiving a particular drug, as opposed to patients recruited to trials. Real world data can enhance patient management because the length of follow-up can be much longer than randomized control trials (RCTs); they include patients with complex issues, of the type seen in routine clinical settings, who are often excluded from RCTs due to the exclusion criteria; and cohort sizes tend to be larger than conventional RCTs. They provide health care professionals and researchers with information on people with diseases, at the individual and group level, often over time enabling analysis of trends in treatment, risks of adverse events (e.g. infection) and disability.

Insight into the outcomes that are linked to particular disease trajectories can be obtained from clinical and demographic factors, disease and treatment history, and co-morbidity and risk exposures that are recorded in real world databases. However, these factors often only partially explain intra-patient differences in important clinical outcomes, suggesting that other factors contribute. Such factors could be biological, many of which can be measured directly or through a proxy; such biomarkers can correlate with stage of disease, response to treatment or adverse events [1]. Biomarkers may include serological, genetic, epigenetic or transcriptomic factors or might be captured in imaging data or in spectral maps of the proteome retrieved from relevant biological samples.

Advantages of biomarkers over routinely collected clinical data are that the markers are measured objectively and therefore not subject to observer bias, they can be measured reliably and precisely, and they are closer to the pathology and may provide mechanistic insight. Some biomarkers become so accepted that they become part of routine data collection; for example, anti-citrullinated peptide antibodies (ACPA) detected by anti-cyclic citrullinated peptide (anti-CCP) antibodies are now routinely tested because they define a subset of cases with RA with a more severe disease course [2].

In this review we will consider the advantages that addition of biomarker data to real world datasets brings, discuss how biomarker data have improved our understanding of disease outcomes in RA, and consider the challenges faced when interpreting biomarker discoveries or when planning to add biomarker data to real world data collections.

## Key challenges that can be addressed using biomarker data

Once a patient presents with inflammatory arthritis, it is now widely accepted that the primary goal should be to control disease activity as quickly as possible. Indeed, real world data have shown that control of disease activity within the first 6 months of presentation of inflammatory arthritis can influence outcome 20 years later [3]. Over the past 20 years, significant advances in the management of RA have resulted in the development of new drugs that target various immune pathways and molecules, including cytokines (tumour necrosis factor (TNF) and interleukin-6), T cell signalling and B cell depletion [4]. Despite the large number of treatments now available to patients, it is still not clear which drug will be of benefit to an individual patient as each drug has a significant non-response rate. The challenge is to identify RA patients as early as possible before structural joint damage has accumulated and give them the most appropriate treatment for their disease. In an ideal world, robust biomarkers would be available to guide clinicians in the need for therapy (some patients will remit without treatment) and the treatments most likely to effectively control inflammation.

## Disease development and progression

Biomarkers have provided a better understanding of the biological pathways underpinning an individual’s risk of developing RA and subsequent disease progression. For example, genetic biomarkers anchor the relationship between the human leucocyte antigen (HLA) gene *HLA*-*DRB1* and RA development [5–8] implicating the activation of CD4-positive T cells as a critical step in the aetiology of the disease [9]. Indeed, large scale genome-wide genetic variant analysis, including over 100 000 individuals, has identified over 100 genetic regions outside the HLA region that are associated with RA development [6, 10] and many of these regions harbour genes that map to T cell activation pathways [11]. In other complex diseases, such as cardiovascular disease, information from variants across the genome has been used to construct genotypic risk scores, which identify those at highest risk of developing cardiovascular disease. Given that cardiovascular disease is common and primary prevention strategies are established, the usefulness of a biomarker score associated with a tripling of risk is potentially clinically useful [12]. However, for RA where the risk of disease is lower, identifying a biomarker associated with a tripling of risk is not clinically useful. Indeed, *HLA-DRB1* genetic variants have long been established to confer that magnitude of risk but are not used to screen the population because carriage of the variants only increases risk from 1% to 3% (i.e. 97% risk of not developing RA).

The severity of joint destruction in RA is influenced by genetic factors, with an estimated heritability of 50% [13]. Some of the intra-patient differences in disease progression are explained by carriage of risk haplotypes at the *HLA-DRB1* locus, which are also strongly associated with RA susceptibility. Importantly, the DRB1 haplotypes that confer the greatest risk of disease development also identify patients with the most severe radiographic progression [14]. However, non-HLA genetic biomarkers of erosive disease have not been convincingly found, probably due to a lack of statistical power to detect subtle genetic effects.

Serological biomarkers of RA development and severity have been recognized for some time. A positive titre for anti-cyclic citrullinated antibody (ACPA) and rheumatoid factor (RF) can precede RA development by several years [15, 16] and are associated with a more severe disease course [2]. Both biomarkers perform individually well at classifying people with RA, compared with healthy controls. For example, in a recent large meta-analysis, the pooled sensitivity for ACPA and RF was 67% and 69% and the pooled specificity was 95% and 85%, respectively [17]. Indeed, ACPA and RF status now form part of the established classification criteria for RA [18] and are important enrichment biomarkers for defining RA cohorts for further study. The presence of other serological changes is also observed in the pre-symptomatic phase of RA; for example, a positive titre for anti-carbamylated (anti-CarP) antibodies precedes RA development in a subset of patients and is associated with a more severe disease course independently of ACPA [19–21].

## Treatment outcomes

Over many years, high quality real world cohorts and registries of patients have been established to explore the long term safety and treatment outcomes for patients receiving medication for their RA. A number of important clinical factors are now known to correlate with clinical response to methotrexate and biologic drugs, including TNFi-inhibitors (TNFi). Gender and pre-treatment disability and activity [22, 23] explain some of the variation in response but these factors are not sufficiently predictive to be useful in a clinical setting, nor do they capture information about relevant target pathways or intra-patients differences in treatment exposure or adherence. Focus has therefore shifted to identifying treatment response biomarkers.

The search for reliable biomarkers of treatment response has led to an enormous effort in establishing large-scale biological sample collection in real world cohorts (Table 1). This has paved the way for analysis of genomic and detailed clinical data [25], as well as large national [30] and international collaborations [31]. For example in the UK, the Medical Research Council and Arthritis Research UK jointly funded the MATURA (MAximising Theraputic Utility for Rheumatoid Arthritis) consortium [30] recognizing the need to maximize the interconnect between specialized groups with diverse expertise such as researchers, statisticians, medical practitioners and industry partners.

**Table 1:** Examples of real world drug registries that include biomarker data in RA

Short name	Country	Type	Reference
BIOBADASER	Spain	Registry	[24]
BSRBR-RA/ BRAGGSS	UK	Registry	[22, 25]
DANBIO	Denmark	Registry	[26]
DREAM	Netherlands	Registry	[27]
SCQM	Switzerland	Registry	[28]
ARTIS	Sweden	Registry	[29]

ARTIS: Antirheumatic Therapies in Sweden; BRAGGSS: biologics in Rheumatoid Arthritis Genetics and Genomics Study Syndicate; BSRBR-RA: British Society for Rheumatology Biologics Register for Rheumatoid Arthritis; DREAM: Dutch Rheumatoid Arthritis Monitoring; SCQM: Swiss Clinical Quality Management in Rheumatic Diseases.

MATURA is still in progress but important findings from this research are beginning to emerge including the development of a classifier that is highly predictive of MTX non-response using genome-wide gene-expression data [32], the identification of genetic markers that are correlated with TNFi [33] and MTX [34] response, and the observation that genetic markers strongly correlate with the objective sub-components of the DAS28 i.e. serum C-reactive protein (CRP) level and the swollen joint count (SJC, out of 28 joints [35]), but not the tender joint count or patient’s assessment of general health; traits that are not heritable are very difficult to model using biological factors such as biomarkers.

### Genetic variants

Numerous genetic biomarkers of poor treatment outcomes in RA have been reported in the literature, but with modest confirmatory evidence between studies. Genetic markers with some evidence of replication in studies of TNFi response include variants at the *PTPRC*, *FCGR2A*, *TRAF1/C5*, *CHUK*, *IRAK3* and *NFKBIB* loci [36, 37]. However, none are sufficiently predictive of response alone to be clinically useful.

As mentioned above, large scale genome-wide genetic variant analyses have identified a great many genetic regions that are associated with RA development [6, 10]. Importantly, numerous genes within identified regions interact, at the protein level, with targets of approved treatments licensed for RA, and for other diseases [6], indicating that robust genetic association studies may identify treatment response subgroups, or help to reveal drug mechanisms [38].

### Epigenetic biomarkers

Epigenetic biomarkers such as DNA methylation and covalent histone modifications regulate gene expression through modulating accessibility of transcription factors to DNA [39]. Epigenetic marks are therefore a potentially important source of biomarkers of treatment response (reviewed elsewhere [39]). For example, the recently reported differential DNA methylation at the *LRPAP1* gene locus on chromosome 4 is correlated with response to the TNFi etanercept. Initially identified in 72 patient samples this finding was supported by genetic validation in a larger collection of 1204 TNFi treated patients [40]. Low density lipoprotein receptor-related protein 1 (LRPAP1) is highly expressed in mononuclear cells [41] and influences activity of transforming growth factor β [42], a potent anti-inflammatory cytokine [43].

To identify biomarkers of MTX response, Carini *et al.* used a recently developed DNA-array-based method to assessed 13 322 potential chromosome interactions relating to 123 genes with known importance to RA [44]. The authors identified a chromatin conformation signature consisting of five genomic regions (genes *FNAR1*, *IL-21R*, *IL-23*, *CXCL13* and *IL-17A*) that was able to detect non-responders to MTX with 90% sensitivity when validated in independent samples. Although further independent validation of this important finding is now required, it is attractive to consider how this biomarker signature/method could be implemented clinically. The method relies on uncomplicated sample processing (a small amount of whole blood is required) and makes use of an established laboratory technique (quantitative polymerase chain reaction).

### Gene expression/transcriptomics

Gene expression profiling studies of TNFi response have reported few differences in pre-treatment samples between future EULAR good and non-responders, whereas marked differences in gene expression profile are observed in good responders when contrasting post-treatment samples with samples collected pre-treatment [33]. Therefore, early rather than pre-treatment biomarker profile may be identified in certain data types.

### Drug levels and antidrug antibodies

Measuring drug levels in TNFi-exposed patients provides information as to whether a therapeutic circulating drug level is achieved [45, 46]; as expected, disease control is sub-optimal in patients with sub-therapeutic drug levels. Factors affecting drug levels include body mass index and presence of antibodies directed against the drug. A recent report [47] suggests that development of anti-TNFi antibodies in patients with Crohn’s disease is associated with genetic markers within the HLA region (specifically HLA-DQA1*05), a finding that now requires replication. Importantly, studies also show that patients with high circulating concentrations of TNFi are at increased risk of developing respiratory and skin infections, compared with patients with low circulating TNFi levels [48], suggesting that monitoring of drug levels provides clinically important information regarding under- or over-dosing patients.

### Multi-omics

To achieve a more complete view of the mechanisms that underpin response to treatment in RA, it is likely that biomarkers present in a number of data types will need to be measured in the same individuals permitting a more holistic analysis approach. Recently a report by Tasaki *et al.* [49] showed the utility in a multi-omics approach to understanding treatment response in RA. By comparing blood transcriptomic, proteomic and immunophenotype profiles between patients, pre-and post-treatment, and healthy controls, the authors identified signatures associated with clinical remission. The authors were also able to make a distinction between clinical remission and molecular remission in patients who experienced successful treatment. Importantly, patients who achieved molecular remission, i.e. a biomarker profile more similar to healthy controls than to RA, achieved better long-term treatment outcomes, particularly if molecular remission was observed across more than one data type [49]. The study was small and the findings require further validation but the research highlights the importance of considering multiple data types as well as on-treatment sampling.

## Challenges faced

It should be recognized that, whilst some clinically useful biomarkers have emerged from studies, many published biomarker studies never translate to the clinical setting for a range of reasons discussed below.

### Logistical challenges

There is a great deal of discussion about storage and transportation of samples prior to biomarker discovery and some recommend that sample collection should follow international recommendations. This is difficult for real world biobanks associated with disease registries as sample collection is often opportunistic, when patients are being seen clinically. Undoubtedly, this could lead to false-positive reports of associations and, therefore, it is vital that positive associations are validated in independent data sets. The clinically useful biomarkers that have emerged, including autoantibodies and some proteomic biomarkers such as CRP, have proved themselves to be robust to collection, storage and transportation issues as they are relatively stable once collected. It is likely that biomarkers that translate successfully to the clinic in the future will either also need to be stable (hence the attraction of genetic variants as potential biomarkers) or be collected in a consistent, reproducible way to avoid generation of misleading data. One way to tackle this in real world data may be to test the stability of potential biomarkers [50] or to use repeated measures to smooth out the natural variation that occurs.

### Limitations of outcome measure

Rather than being useful for prediction, assessment of on-treatment biomarker levels may provide a quantifiable measure for monitoring disease. Indeed serum CRP and the erythrocyte sedimentation rate (ESR), included in the DAS28, and the multi-biomarker disease activity (MBDA) score are already used to monitor disease activity [51, 52]. However, none are specific for RA and only correlate moderately well with synovitis [52, 53] or long-term outcomes [54].

The DAS28 encompasses both objective [SJC, CRP (or ESR)] and subjective (tender joint count and patient’s assessment of general health) measures, the latter scores receiving higher weightings [51]. We have previously reported that psychological factors, such as depression and illness beliefs, correlate more closely with the subjective components than with the objective components [55] suggesting that the DAS28 also measures factors other than synovial inflammation, although the latter is the primary target for treatment. This finding has added to the debate of what we mean by ‘treatment response’. There is an argument that, as many drugs were developed to specifically treat joint inflammation, whether an individual is classified as a responder should be based on how well joint inflammation is controlled. One recent study found that only the CRP and SJC were correlated with ultrasound-observed synovitis and a re-weighted score including only those components was better correlated with radiographic progression, compared with the DAS28 composite score or its individual sub-components [56].

Psychological factors, including high pre-treatment anxiety scores, have been reported to be associated with non-response to methotrexate, with the definition of non-response including patients who stopped their treatment before 6 months [23]. However, it is not clear whether the drug was effective at controlling synovitis; going forward, separating the concept of overall treatment response and effective control of synovitis will increase the likelihood of useful biomarkers being identified for the latter as biomarkers are more likely to reflect a biologically rather than psychologically driven process.

### Inadequate adherence to treatment

Even with an optimized treatment response outcome that is reflective of synovial inflammation, the identification of biomarkers predictive of response would still not be straightforward as a number of factors can confound response prediction. We have previously reported that self-reported adherence to TNFi therapies was sub-optimal (27% of patients self-reporting non-adherence) and correlated with future response [58]. Therefore patients predicted to respond may be classified as non-responders because of non-adherence, reducing the accuracy and power of predictive biomarker studies. Inadequate adherence can be measured or monitored using appropriate biomarkers. For example, an HPLC–mass spectrometry assay for monitoring low dose MTX and its major metabolite, 7-OH-MTX, in urine samples from RA patients has recently been developed [59]. The assay can detect MTX for up to 105 h after administration and 7-OH-MTX for up to 98 h, suggesting that this platform is suitable for assessing adherence to therapy in a clinical setting.

### Lack of comparison group

RA is a relapsing–remitting disease in which patients experience moments of high and low disease activity as part of the natural disease course. Therefore a distinction that is difficult to make in real world datasets is whether disease activity has improved because of successful treatment, or because disease activity was initially very high and has more room to improve before the subsequent clinical visit. In a trial setting the issue is resolved by randomizing patients to one of two (or more) treatment arms but that is not possible in the real world. In statistics the phenomenon is referred to as regression towards the mean and must be considered when designing biomarker studies or when interpreting results from real world datasets. Ensuring matching of the baseline characteristics of the two comparator groups can be used for within-cohort studies or propensity score matching [60] can be used otherwise.

### Specificity

Once biomarkers of clinical response to treatment have been identified, the next challenge will be to test if they are predictive of response to a specific treatment or if they are specific to a particular disease. Here numerous datasets are required, including patients exposed to alternative treatments, across different stages of disease and across different inflammatory diseases. In the UK, these datasets have already been established in highly successful national stratified medicine programmes in RA, psoriasis, systemic lupus erythematosus, Sjogren's syndrome, autoimmune hepatitis and primary biliary cirrhosis. The Immune-Mediated Inflammatory Disease Biobanks in the UK (IMID-Bio-UK) [61] is bringing together these datasets with the aim of harnessing biological samples, deeply phenotyped clinical cohorts and high quality biomarker data to address related and overlapping precision medicine questions that cannot be addressed exclusively in the individual collections.

### Statistical challenges

Analysing real world databases, with or without biomarker data, exposes a number of potential biases that require consideration. A recent editorial published in Nature has highlighted statistical challenges that should be considered in designing and analysing studies. These include regression to the mean, natural variation, the selection of an appropriate outcome measure and using a continuous rather than a dichotomous outcome. Some of these issues are discussed in more detail in the review by Prof. Til Sturmer as part of this supplementary edition.

### Interpreting findings from biomarker studies

A large number of biomarker studies have been published yet few markers have reached the clinic. When interpreting the findings of biomarker studies, therefore, a degree of scepticism is healthy. Validation of findings is a pre-requisite but even if replicated in independent datasets, a biomarker may not add sufficient information to make it clinically useful. How predictive a biomarker needs to be to be clinically useful will depend very much on the context in which it is used. For example, diagnostic accuracy is very important for tests that will determine whether treatment is given or withheld but a test with less stringent performance characteristics may be adequate where choices are being made between treatments, i.e. preferential prescribing (complementary diagnostics). Several studies are underway currently to establish recommended cut-offs for tests aimed at guiding selection of the first biologic therapy for RA, for example. Demonstration of clinical utility may need to be followed by health economic assessment to show that the cost of the test does not outweigh the benefit accrued. Helpfully, the OMERACT Consortium has developed guidelines for the clinical validation of biomarkers [50].

## Summary and future perspective

In summary, sceptics would argue that, despite huge investment in biomarker research, few successes have emerged. We would argue that biomarker studies have added value to real world data collections in inflammatory arthritis and have already identified biomarkers that are being used or have the potential to be used in routine clinical practice. These include anti-CCP testing, which is already in use; drug level and antidrug antibody testing in patients being treated with TNFi, which is currently being reviewed by NICE; and anti-CarP antibody testing, which requires further assessment of clinical utility. A major goal for biomarker research is to help inform and personalize the choice of treatment recommended to patients to maximize clinical benefit. Before this goal can be realized in RA, a quantitative disease activity measure(s), which is easy to measure for all patients and is reflective of synovial inflammation, is first needed and will likely be discovered by well-designed biomarker experiments. For example, assessing blood-based biomarkers for correlation with MRI-observed synovitis might identify a biological surrogate for active joint-specific inflammation that is better than CRP alone and can be objectively measured in all patients. Other major obstacles to overcome include the inadequate control of confounding, which can partly be addressed by testing for drug adherence and anti-drug antibody titres and by more sophisticated methods of matching patient groups for comparison. Reproducibility of biomarker discoveries to date has been hampered by a lack of statistical power that can partly be addressed by increased funding for establishing and maintaining real world datasets and collection of biological samples as part of expected practice. Discovery of biomarkers of clinical importance will also likely require large collaborative efforts involving many academic groups and industry partners. The emergence of high dimensional datasets and advanced data-driven statistical methods, such as machine learning, offer the potential to develop accurate, robust and discriminative statistical classifiers of important clinical outcomes in RA. It is possible that many small differences can be combined to develop scores that will, together, allow better prediction of clinically important outcomes; for example, genotypic risk scores have been proposed to aid identification of patients at the highest risk of developing cardiovascular disease [12]. However, underpowered and unreplicated studies will potentially suffer from over-fitting and poor model performance so any models developed need to be tested in a prospective clinical setting.

*Funding:* D.P. is funded by the NIHR Manchester BRC. We acknowledge support from Arthritis Research UK (grant ref 21754) and MRC/Arthritis Research UK stratified medicine award, MATURA (grant ref MR/K015346/1).

*Disclosure statement*: The authors have declared no conflicts of interest.
